# Response analysis of the nodes of pipe networks under seismic load

**DOI:** 10.1371/journal.pone.0247677

**Published:** 2021-03-08

**Authors:** Delong Huang, Aiping Tang, Qiang Liu, Dianrui Mu, Yan Ding

**Affiliations:** 1 School of Civil Engineering, Harbin Institute of Technology, Harbin, Heilongjiang Province, China; 2 School of Mechanical and Electrical Engineering, Harbin Engineering University, Harbin, Heilongjiang Province, China; China University of Mining and Technology, CHINA

## Abstract

Transient ground displacement (TGD) that is caused by earthquakes can damage underground pipes. This damage is especially critical for the joints, elbows and tees of the pipes which play an important role in the operation of a pipe network. In this study, a scale pipe network with both elbows and tees, as well as some components of the pipe network with only tees or elbows, has been investigated. The response of the nodes of a pipe network, when installed in non-uniform geology, was analyzed using the shaking table test and ABAQUS finite element simulation. This paper has firstly introduced the preparation of the test and the developed finite element model. Then the system response in terms of strain, the friction, the bending deformation, the node deformation amplification coefficient and the pipe-soil relative displacement along the pipe axis of the pipe network and two pipe network components have been analyzed explaining the correlation between these responses. Finally, the influence of elbows and tees on the pipe network was analyzed, and the conclusions that have been reached about how tees and elbows can change the response of a pipe network during an earthquake can provide theoretical support for the seismic design and layout of an underground pipe network.

## Introduction

**Fig 1:**
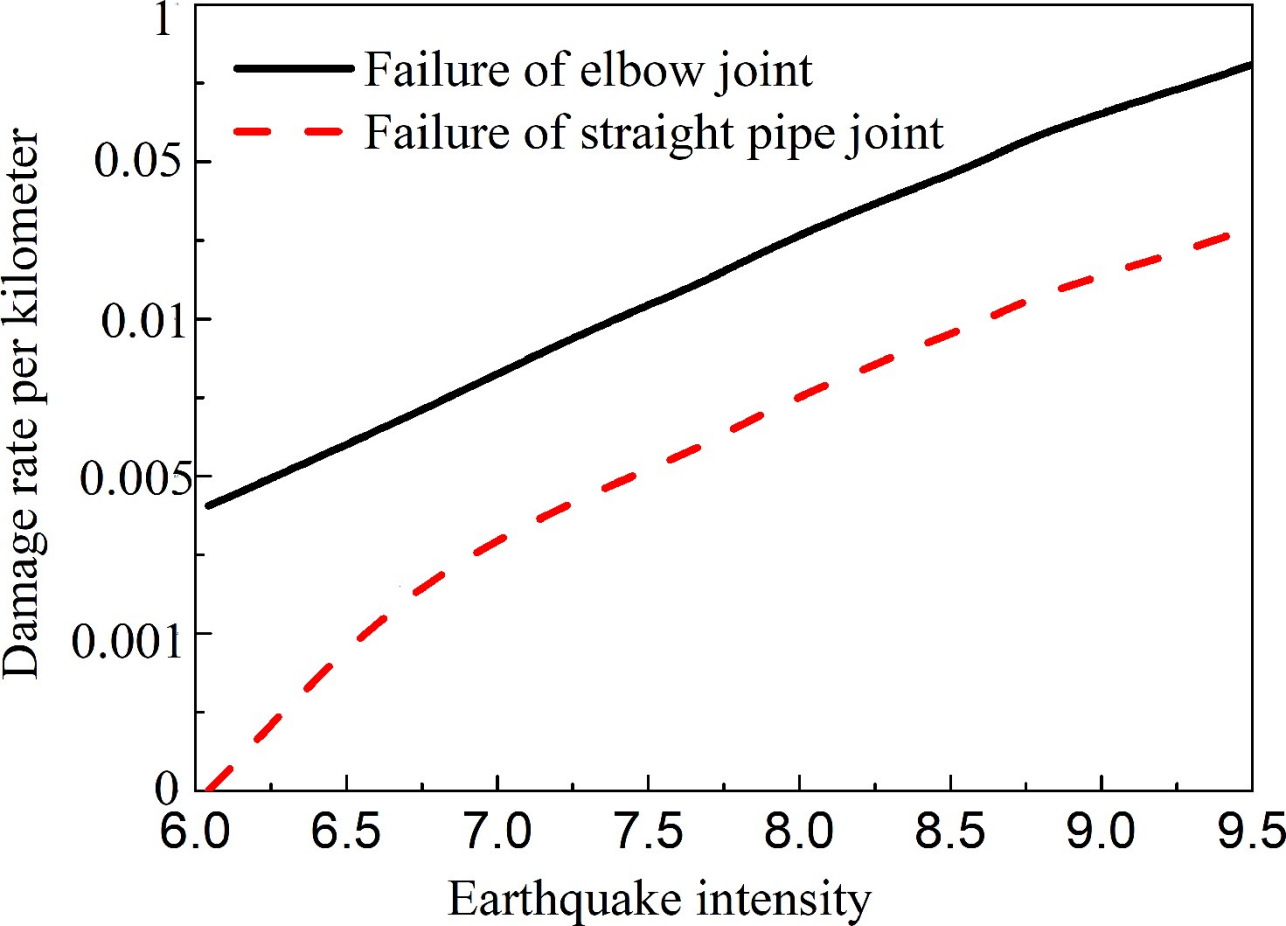
Comparison of joint vulnerability between elbow and straight pipe [2].

Transient ground displacement (TGD), which is caused by seismic waves, is a type of geological hazard that can be triggered by earthquakes. The damage to the joints of pipe networks and their components, such as elbows and tees, is the main form of damage that can occur as a result of an earthquake [1]. Fig 1 displays a summary of the vulnerability of pipe networks to previous seismic damage [2].

The water supply and the gas supply network are an important part of the urban lifeline system. In many earthquake investigations, it was found that damage to an underground network may cut off the supply of drinking water, fire hydrants and gas, and can cause secondary disasters such as urban waterlogging and fire, resulting in a huge loss of life and property [3]. In 1995, during the Osaka Kobe earthquake (M = 7.3), there were more than 20,000 leaks from gas pipes in the Osaka area, and most of them occurred at the elbows of welded steel pipes. For the water supply pipes, 900,000 households were cut off from the water supply, 23 nodes, such as elbows, were damaged near the Kanzaki River, and the sewage and rainwater drainage networks in Kobe City were also severely damaged, covering an area of nearly 3m/ha [4]; In 2008, during the Wenchuan earthquake (M = 8.0), nearly 20 cities and counties in Sichuan Province alone, including the underground pipe network, suffered serious damage to the water supply and the gas supply facilities, including gas companies and water plants in Beichuan, Dujiangyan, Mianzhu and other places; although they could operate normally, the underground pipe network was seriously damaged [5,6]. The leakage at the pipe network joints and other important nodes is huge in such a scenario, which then cannot meet the requirements of use, and it is difficult to repair the damage quickly [7]. Therefore, in the seismic design, the urban lifeline pipe network system is the key protection object, in order to ensure rapid repair and normal operation after the earthquake.

At present, research on the underground pipe network system is limited to two aspects, involving: 1) component performance, for which the soil-structure interaction under earthquake loading is evaluated, and 2) system performance, for which the integrated behavior of the network is assessed. These two aspects are very different and are governed by issues that are related to the level of detail that can be achieved in the component vs. system characterization, as well as the spatial variability, uncertainties in the properties of the materials and the state of repair of the components, the network’s flow laws, and the operational logic of the system [8]. For example, these researches had been done by scholars [9–13]. They only focused on the failure probability of the pipe network under seismic load, and they did not study the mechanical mechanism of the failure of the pipe network. Therefore, the question of how to apply the seismic response of simple pipe components in the performance analysis of a complex pipe network is an important aspect of the current pipe network system research.

The study into the mechanical research methods of an underground pipe during an earthquake includes numerical analysis, theoretical calculation and experimental research currently. For numerical analysis, most scholars use the finite element method [14–22]. One common point of these studies is that scholars only focus on a single type of straight pipe, but not on elbows and tees of the pipe network. Until Liu et al. [23] conducted a numerical study on the response mechanism of an underground pipe network by using a test with an artificial explosion. Although the test is a full-scale model, the accuracy of using explosion to simulate earthquake load needs to be studied. For the theoretical calculation method of underground pipe, Qu et al. [24] used the random vibration theory to calculate the longitudinal and transverse seismic response of pipe; Chakraborty and Kumar [25] used the theoretical analysis to study the vertical pull-out force on pipe in sand; Almahakeri et al. [26] studied the vulnerability calculation of natural gas pipe under the seismic effect. Although it is common to study underground straight pipes with analytical methods, a large number of simplifications and assumptions are needed [27]. Therefore, for the analysis of pipe network, it is necessary to assume many ideal situations, and these assumptions need to be verified by dynamic tests. Therefore the most effective way to study the seismic mechanical response of pipe network is the experimental research. The shaking table test and centrifuge test are the effective methods to apply dynamic loading on the pipe-soil system. These shaking table test studies are based on the single type of straight pipe. For example, Meng et al. [28] inserted the steel pipe into two laminated shear boxes fixed on two different shaking tables to test the response of pipe under the excitation of non-uniform seismic wave. Sim et al. [29] tested the dynamic behavior of acrylic acid pipe crossing vertical fault with shaking table. Yan et al. [30] studied the response of underground pipe under three-dimensional non-uniform seismic excitation by the shaking table test. Due to the limitation of the size of the shaking table, the shaking table test for the pipe network is still limited. Only Wang et al. [31] and Liu et al. [32] had made a 24×24 m pipe network, which used the method of artificial explosion to simulate the seismic source, but this method was only a rough simulation of the earthquake and unable to accurately determine the magnitude and intensity of the earthquake.

Based on the above analysis, this paper has studied the seismic response of an underground pipe network by means of the shaking table test. The specific research process was as follows:

- Straight pipes, elbows and tees made from PVC-U (Unplasticized polyvinyl chloride) were used to make the scale pipe network as well as H-shaped and Z-shaped pipe network components. According to the research content, the corresponding sensors were laid. The H-shaped and Z-shaped pipe networks were put into the soil of the shaking table, and the same artificial waves were then applied;
- Based on the shaking table test, three-dimensional finite element models of scale pipe network, H-shaped and Z-shaped pipe were established;
- According to the axial strain of the scale pipe network in the direction of the seismic wave, the friction force, the bending deformation, the deformation amplification coefficient of the nodes and the pipe-soil relative displacement in the corresponding direction of the pipe were analyzed;
- Then the test results of the axial strain of the H-shaped and Z-shaped pipes were analyzed, and finally the seismic response relationship between the nodes of the buried pipe network was determined.

## The design of the shaking table test

### The pipe

**Fig 2:**
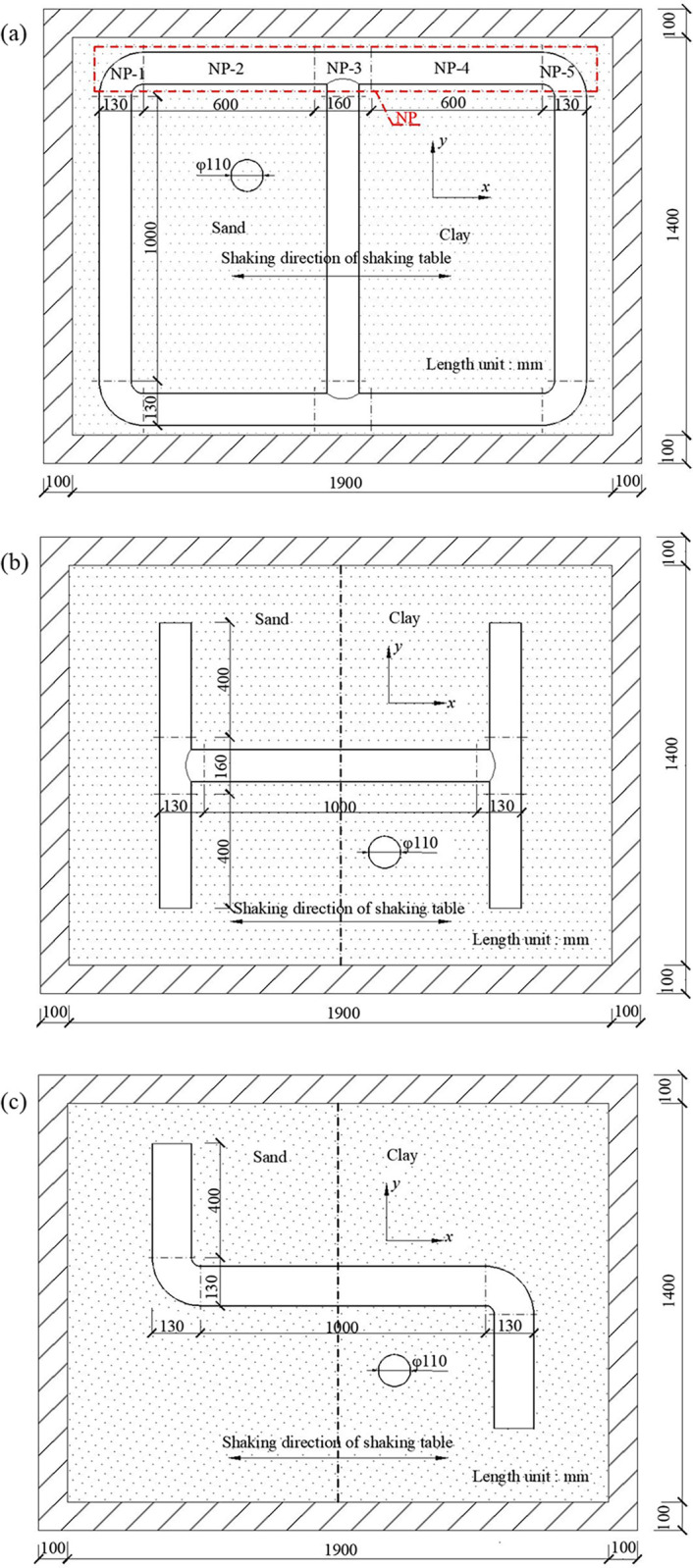
Form and layout of pipes for test. (a) Scale pipe network, (b) H-shaped pipe, (c) Z-shaped pipe.

PVC-U is a type of hard-plastic; based on the influence of the scale of the model, the seismic waves could not destroy the pipe since it was in its elastic stage. Therefore, the internal forces and the deformation tendency of the PVC-U pipes were the same as that of iron pipes. Therefore, because of their similar elastic modulus relationship, PVC-U pipes could be used to simulate iron pipes. Fig 2A has shown the layout and the size of the pipe network; the internal dimensions of the shear box were 1900 mm×1400 mm. From the corresponding measurements, these pipes had a diameter of 4 inches (110 mm), a wall thickness of 3.2 mm, an elastic modulus of 4000 MPa, a density of 1400 kg/m^3^, a Poisson’s ratio of 0.3 and a buried depth of 0.3 m. The actual engineering buried depth of the network was 3.0 m through the similarity ratio conversion. By inserting and applying glue to the elbow of the pipes in order to simulate the welding of the iron pipe’s elbows and tees, the displacement of the pipe network’s nodes could be determined by the pulling distance of the elbow or tee. For the construction of the pipe network, the elbows and tees were combined to form a scale pipe network to study the axial response of the pipe. As there are both tees and elbows in the pipe network, a single type of pipe joint was established to study the seismic response. In this study, H-shaped and Z-shaped basic node forms were constructed to analyze the influence of tees and elbows on the pipe network during an earthquake. The pipe’s size and its position in the shear box are shown in Fig 2B and 2C.

### The sand and clay

**Fig 3:**
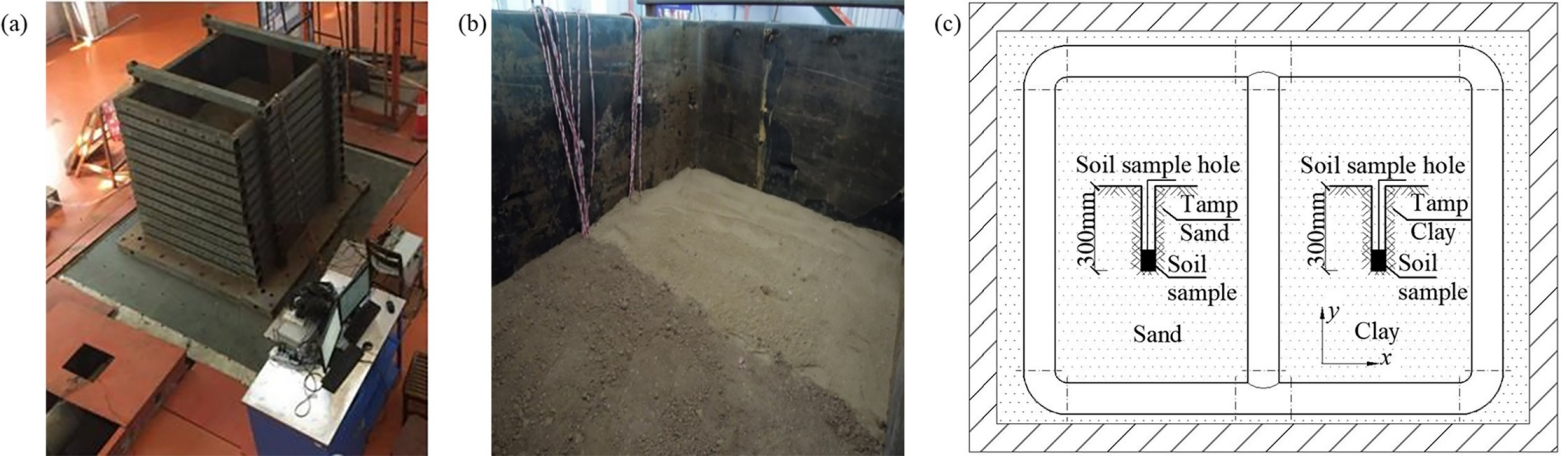
Preparation of test soil and preparation of soil sampling for soil parameters. (a) Laminar shear box, (b) The division of sand and clay, (c) Schematic diagram of soil sampling.

In the test, the sand and clay were packed into the laminar shear box on either side of the pipe and tamped down layer by layer. 2cm thick rubber was embedded in the inner wall of the shear box in order to prevent soil leakage and reduce the impact of the shear box on the pipe’s response. The shear box was as shown in Fig 3A, and the division of the sand and clay in the test was as shown in Fig 3B. The compaction height of each layer of soil was 15 cm and the density of the fill was controlled by calculating the volume and mass for each layer of soil. Since the soil was slightly dense, the shear wave velocity of the soil was about 200 m/s; this was calculated from the state and the properties of the soil. After tamping, a sample of the soil was taken from the shear box as shown in Fig 3C, and a ring knife and a pycnometer were used to measure the physical parameters of the soil. For the sand, the following parameter values were measured: density *ρ* = 1692 kg/m^3^, water content *ω* = 0.18%, specific gravity *G*_*s*_ = 2.62, cohesion *c* = 0 and the internal friction angle *φ* = 32°. The corresponding values for the clay were: density *ρ* = 1430 kg/m^3^, water content *ω* = 10.65%, specific gravity *G*_*s*_ = 2.45, cohesion *c* = 5 kPa and the angle of internal friction *φ* = 20°.

### The sensors

**Fig 4:**
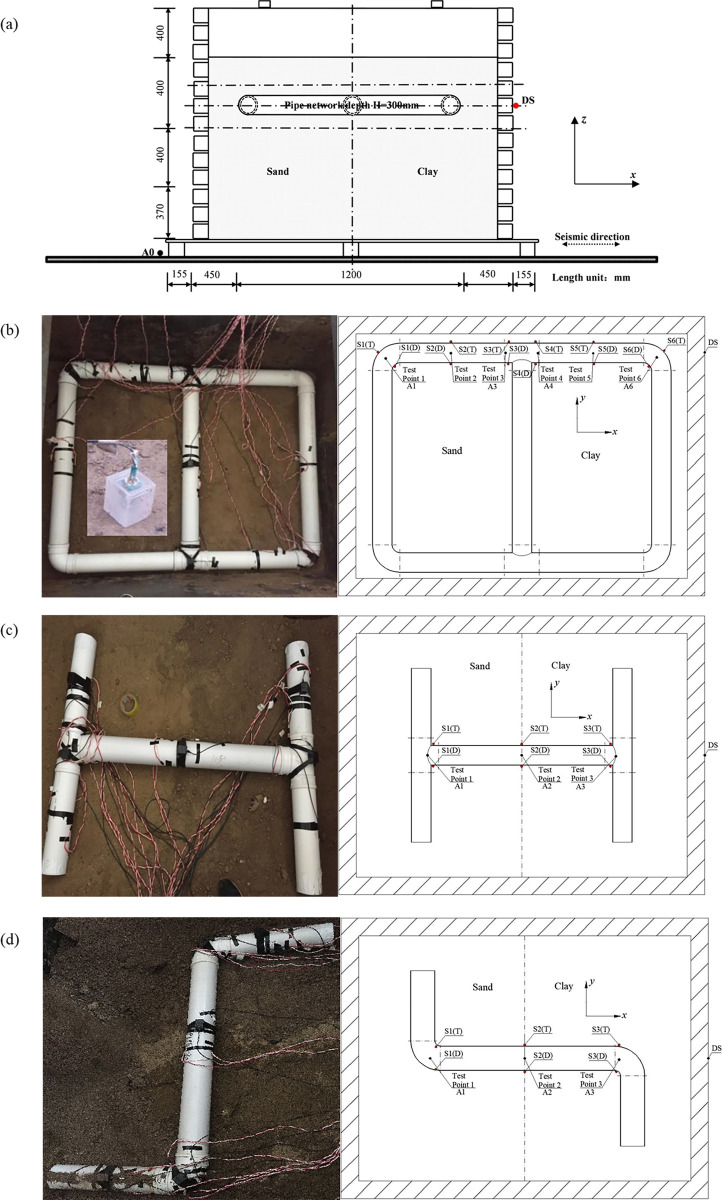
Location of sensors layout for pipe network and two pipe network components. (a) Section of the shear box and pipe location, (b) Sensors layout of pipe network, (c) Sensors layout for tee component, (d) Sensors layout for elbow component.

A LVDT (Linear Variable Differential Transformer) displacement sensor was used in this test; it has an accuracy of 0.1mm. As this kind of displacement sensor can only measure the linear displacement in a single direction on a solid surface, it was located on the outer surface of the shear box, and the arrangement position was buried in the same horizontal plane as the pipe network, which is represented by DS. As the soil was bounded by the soil box, the displacement of the soil was approximately the same as that of the shear box at the same height. Therefore, the displacement sensor DS could be used to measure the displacement of the soil around the pipe network, as shown in Fig 4A, and the German IMC (Integrated Measurement & Control) data acquisition system was used to collect the displacement data, with a sampling frequency of 1000 Hz.

Uniaxial strain gauges were used to measure the strain, and the effect of temperature was eliminated by using temperature compensated strain gauges; the accuracy of the gauges was 0.5με. An IMC data acquisition system was used to measure the strain and the displacement measurement at the same time. The strain gauges were placed on the outer surface of the pipes, and special glue was applied to the outer surface of the strain gauges to prevent the strain gauges from being soaked by the water in the soil, as the pipe was buried in the soil. The location of the strain gauges in the pipe network has been shown in Fig 4B for six sections of S1(T)/(D)~S6(T)/(D). These sensors were arranged in sections in the direction of propagation of the seismic waves, this was because the axial strain of the pipe in the input direction of the seismic waves was the most obvious under seismic conditions [33]. In order to study the axial deformation mechanism of the pipe in the direction of the seismic wave, earth pressure and acceleration sensors were also arranged in these six sections. In this paper, the pipe involved in the pipe network measurement was designated as NP, which included two straight pipes NP-2 and NP-4, two elbows NP-1 and NP-5, and a tee NP-3, as shown in Fig 2A. In order to measure the axial strain, the average value of two points measured at the same pipe section was taken. For example, S1(T) and S1(D) were the upper and lower measuring points for the horizontal plane of the first section.

The arrangement of the sensors for the pipe network’s tee and elbow components has been shown in Fig 4C and 4D. Since there were no nodes in the middle pipe of these two components, only three measurement sections were used.

### The design of the experimental similarity ratio

The expression of the uniform similarity law was the same as that of the dimensional analysis method used to determine the similarity relationship. However, the equivalent density, after considering the additional part of the artificial mass, was needed to determine the mass density similarity ratio, which is also called the lacking artificial mass model. This model only adds part of the artificial mass and ignores part of the acceleration due to gravity, so there is only a certain degree of the gravity distortion effect. In the shaking table test for seismic simulation, and considering the dynamic response of the pipe network nodes, it was very difficult to ensure that the PVC-U pipe system and the prototype iron pipeline fully met the similarity relationship, which was often impossible to achieve under the existing conditions [34]. In this study, the lacking artificial mass model was used to determine the similarity relationship in the tests, according to the characteristics of the dynamic problems. This test can determine the basic principle of the similar design of the shaking table test according to the response mechanism of the pipe network’s nodes, which can be seen in the literature [2]. The similar ratio design of this test has been shown in Tables 1 and 2.

**Table 1:** Similar to the lacking artificial mass model.

Physical quantity type	Physical quantities	Similarity ratio
**Basic physical quantities**	Length *l*	1/10
	Elastic modulus *E*	1/50
	Acceleration *a*	2
**Major perived quantities**	Equivalent density *ρ*	1/10
	Gravity acceleration *g*	Ignore
**Model features**	Model type	Short of the artificial quality
	Counterweight	Partial counterweight
	Distortion physical quantity	*g*

**Table 2:** Shaking table experimental design for the lacking artificial mass similarity ratio.

Groups	Physical parameters	Similar relations	Similar ratio
**Geometric parameters**	Length *l*	*S*_*l*_	0.10
	Area *A*	$S_{A}=S_{l}^{2}$	0.01
	Linear displacement *u*	*S*_*u*_ = *S*_*l*_	0.10
	Angular displacement *θ*	*S*_*θ*_ = *S*_*σ*_/*S*_*E*_	1.00
**Material properties**	Elastic Modulus *E*	*S*_*E*_	0.02
	Stress *σ*	*S*_*σ*_ = *S*_*E*_	0.02
	Strain *ε*	*S*_*ε*_ = 1	1.00
	density *ρ*	*S*_*ρ*_ = *S*_*E*_/(*S*_*l*_*S*_*a*_)	0.10
	quality *m*	$S_{m}=S_{\sigma }S_{l}^{2}/S_{a}$	1.00×10^−4^
**Load performance**	Concentrated force *F*	$S_{F}=S_{E}S_{l}^{2}$	2.00×10^−4^
	Linear load *q*	*S*_*q*_ = *S*_*E*_*S*_*l*_	2.00×10^−3^
	Surface load *p*	*S*_*p*_ = *S*_*E*_	0.02
	Moment *M*	$S_{M}=S_{E}S_{l}^{3}$	2.00×10^−5^
**Dynamic characteristics**	Time *t*	$S_{t}=S_{l}^{0.5}S_{a}^{-0.5}$	0.22
	Frequency *f*	$S_{f}=S_{l}^{-0.5}S_{a}^{0.5}$	4.47
	Velocity *v*	$S_{v}=S_{l}^{0.5}S_{a}^{0.5}$	0.45
	Damping *c*	$S_{c}=S_{\sigma }S_{l}^{1.5}S_{a}^{-0.5}$	4.47×10^−4^
	Acceleration *a*	*S*_*a*_	2.00

### Test seismic wave

**Fig 5:**
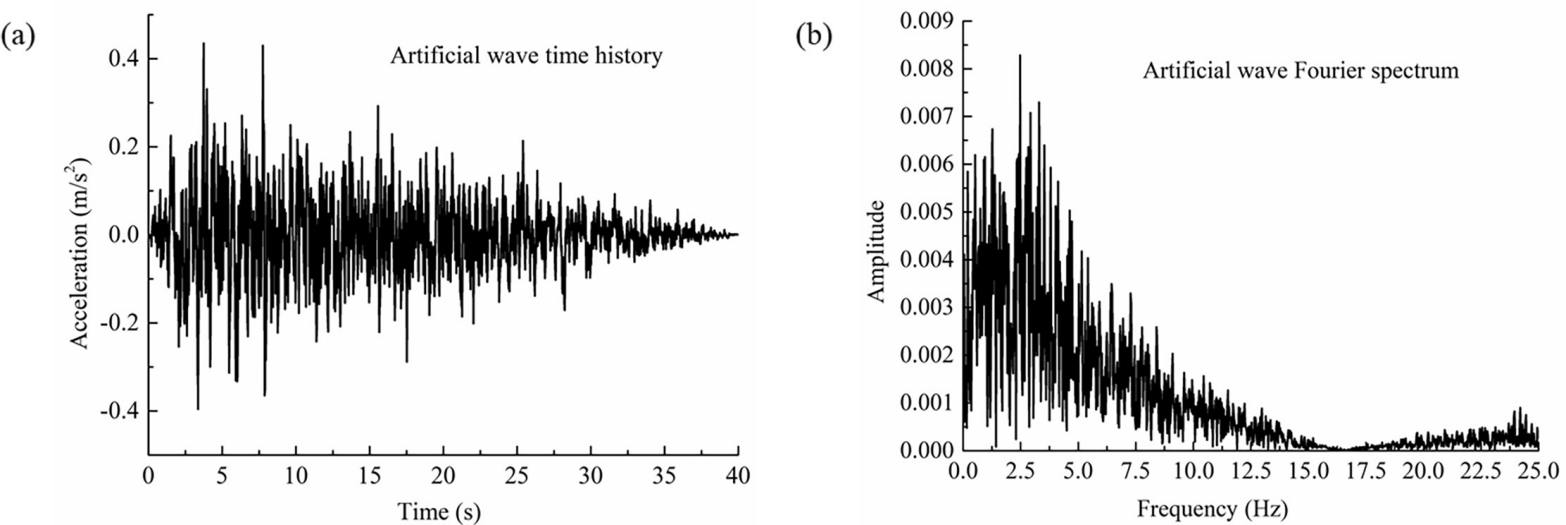
Time history and Fourier curve of seismic wave for test loading. (a) Time history curve of seismic wave, (b) Fourier spectrum curve of seismic wave.

Due to the limitation of the shaking table test, the seismic wave load only acted in a one-dimensional horizontal motion; the most unfavorable seismic wave input was considered in the test design. An artificial wave was selected as the input prototype wave, as shown in Fig 5A. The artificial wave was synthesized using the probability method, with a return period of 50 years, a duration of 70 s, a maximum period of 4 s and a minimum period of 0.01 s. The predominant frequency of the corresponding Fourier spectrum curve was 4 Hz, as shown in Fig 5B. Due to the relationship of the test similarity ratio, the shape of the seismic wave inputted to the shaking table had to be adjusted according to the similarity condition in order to ensure that the wave was compressed on the time coordinate. In the earthquake excitation test in this paper, the peak input value was 0.8*g*. The actual peak value was 0.4*g* through the similarity ratio conversion, which was equivalent to a ground motion with an actual intensity of 9 degrees [35].

### Finite element model of shaking table test

**Fig 6:**
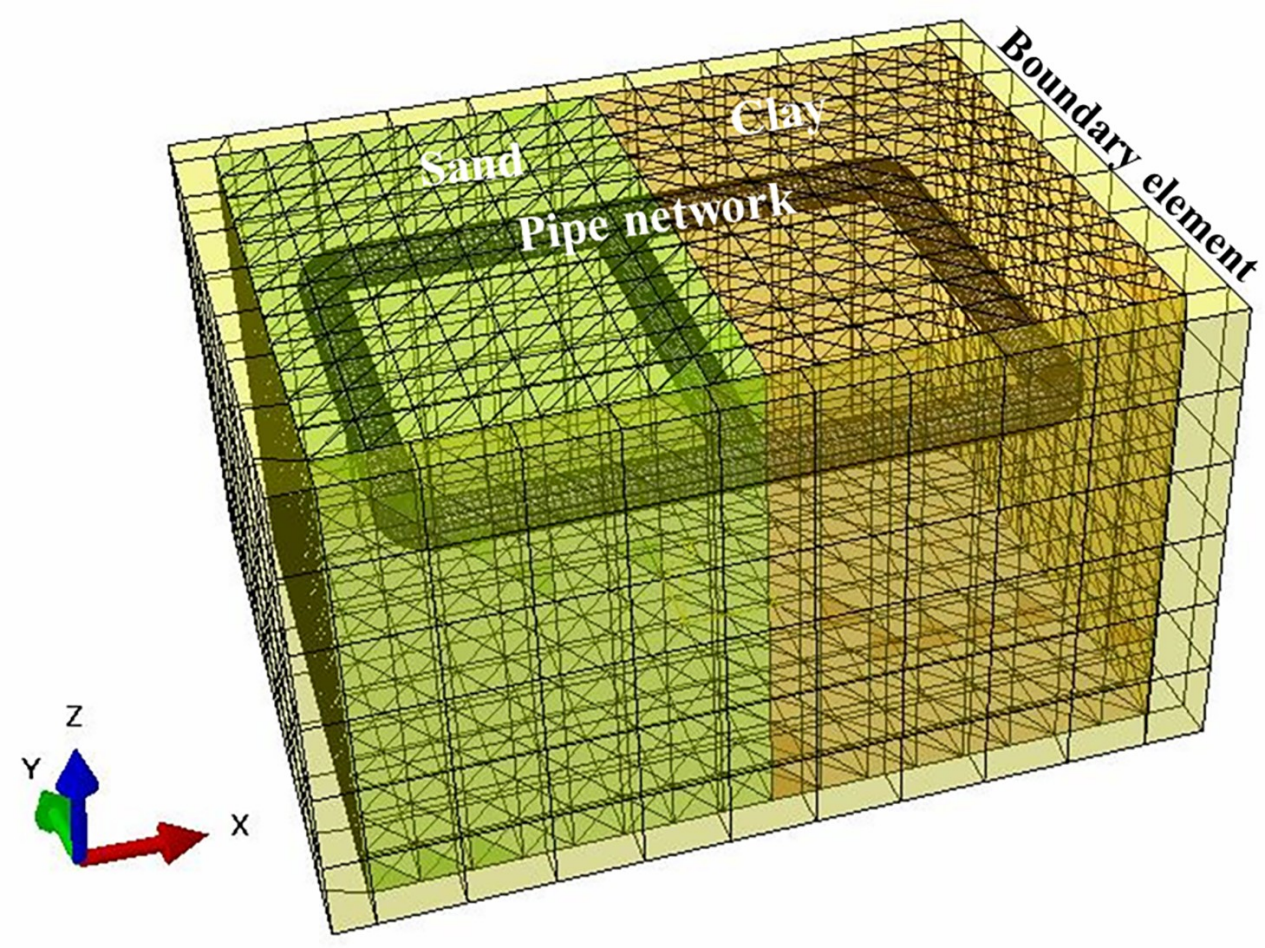
Finite element model of scale pipe network test.

In this study, ABAQUS, a three-dimensional finite element program, was used to compare with the test results, and the responses of pipe network nodes, tee and elbow under seismic action were analyzed. A three-dimensional finite element was developed considering the geometry and configuration of the experimental setup. The finite element model of a scaled pipe network sample, i.e., pipe network, sand, clay and viscoelastic boundaries, as shown in Fig 6.

The constitutive relations of soil and pipe were mainly based on Mohr-Coulomb model and elastic-plastic constitutive models. The material properties and stress-strain curve for the API 5LB was applied to the pipe model [36]. The constitutive model for sand and clay can be described based on the Mohr-Coulomb criterion. This is an elasto-plastic model that uses a yield function of the Mohr-Coulomb form, where the yield function includes the hardening/softening of the isotropic cohesion. On the other hand, the model used a flow potential with a hyperbolic shape in the meridional stress plane and no corners in the deviatoric stress space. This flow potential is thus completely smooth and provides a unique definition of the plastic flow direction. The model parameters of the pipe and soil were the same as those of the test.

The boundary of the model was considered to be viscoelastic [37]. Pipe was simulated by a shell model. Soil medium was discretized by regular hexahedral elements of C3D8R. The seismic wave loaded on the model adopted the same artificial wave as the test, and the peak value was 0.8*g*. Each condition for the implicit method used in the dynamic analysis was determined as follows.

**Fig 7:**
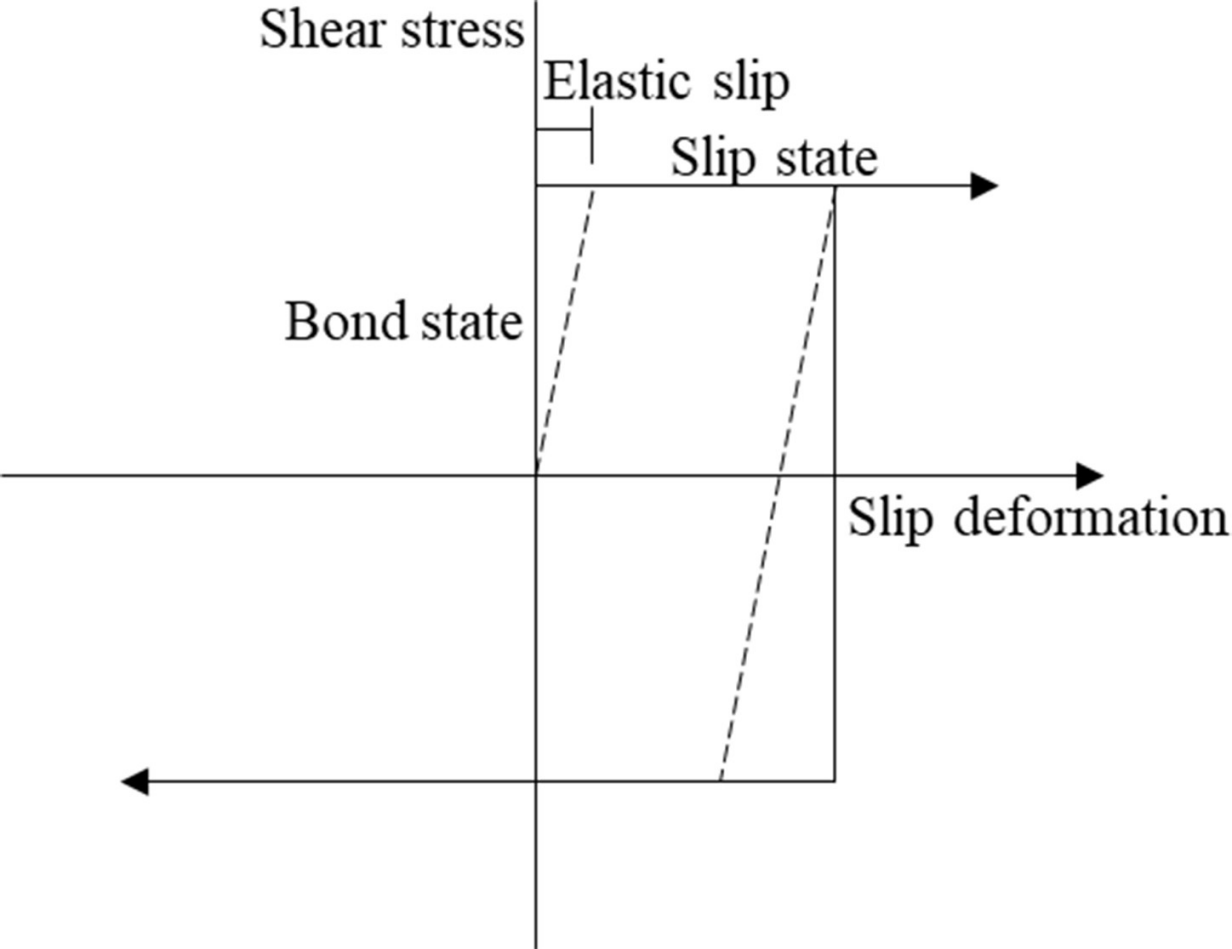
Relationship between deformation and stress of friction contact model.

A contact condition has various contact models, and of them, the shear friction model was the representative contact model that has been used herein. Normal contact was set to be hard-like contact and the tangential contact surface could only transmit tangential stress (i.e., friction), as shown in Fig 7. It has been shown that [38] the choice of the friction coefficient at the pipe-soil interface is related to the roughness of the pipe wall, the type of soil as well as the physical and mechanical properties of the soil. When the pipe’s friction coefficient was 0.4, the shear stress, the axial force and the hoop strain were the smallest and the seismic performance of pipe was the highest. Xiao and Huang [39] studied the friction coefficient between an oil pipe and soil. When the soil was squeezed on a polyethylene surface, and the water content of the sand was 0~16%, it was found that the friction coefficient was between 0.4~0.6. When the water content of the clay was 10%~50%, the friction coefficient was between 0.37~1.10. Therefore, for the shaking table test mentioned above, the friction coefficient of the sand and the clay was about 0.4. In the finite element model, the tangential friction coefficient was also set as 0.4.

## Test results and analysis of the scale pipe network

### Strain test results of the pipe network

**Fig 8:**
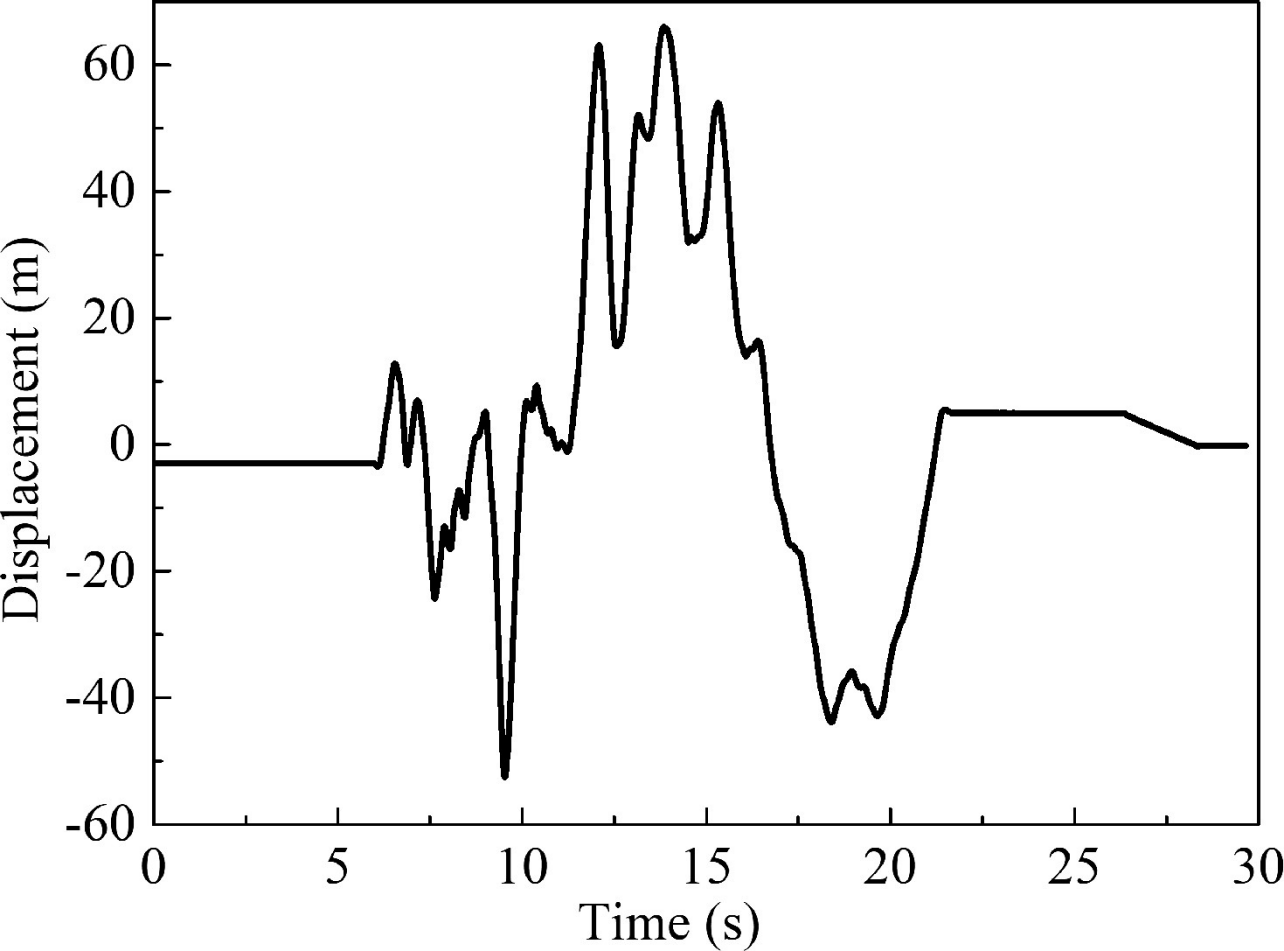
Time history curve of soil displacement outside the pipe network.

The maximum value of the soil displacement sensor DS in the shear box of the shaking table was 65.95 mm, and the corresponding time of the maximum displacement was 13.837 s, as shown in Fig 8. Since the IMC system was used to measure both the strain and the displacement at the same time, the selection time corresponding to the strain was also 13.837 s.

For a selection time of 13.837 s, the strain value measured by the strain sensor at the measuring point of the pipe network NP has been shown in Table 3. The calculated axial strain of the corresponding measuring point was given as:

$$\varepsilon_{x-n}=\frac{\varepsilon_{T-n}+\varepsilon_{D-n}}{2}$$ (1)

where *ε*_*T*-*n*_ is the measured strain values of S1(T)~S6(T), and *ε*_*D*-*n*_ is the measured strain values of S1(D)~S6(D).

**Table 3:** Measured value of strain at each point and calculated value of axial strain.

Strain sensor number	Measured value (με)		Test point	Coordinate (m)	Axial strain (με)	
	Test	Simulation			Test	Simulation
**S1(T)**	-55	-45	Test Point 1	-0.74	-42	-26
**S1(D)**	-28	-20				
**S2(T)**	3	10	Test Point 2	-0.38	-28597	-15308
**S2(D)**	-57197	-30625				
**S3(T)**	-40	-56	Test Point 3	-0.05	-5	-9
**S3(D)**	31	39				
**S4(T)**	30	68	Test Point 4	0.05	14	27
**S4(D)**	-2	-15				
**S5(T)**	18132	15612	Test Point 5	0.38	9063	7799
**S5(D)**	-7	-14				
**S6(T)**	612	314	Test Point 6	0.74	311	166
**S6(D)**	9	18				

**Fig 9:**
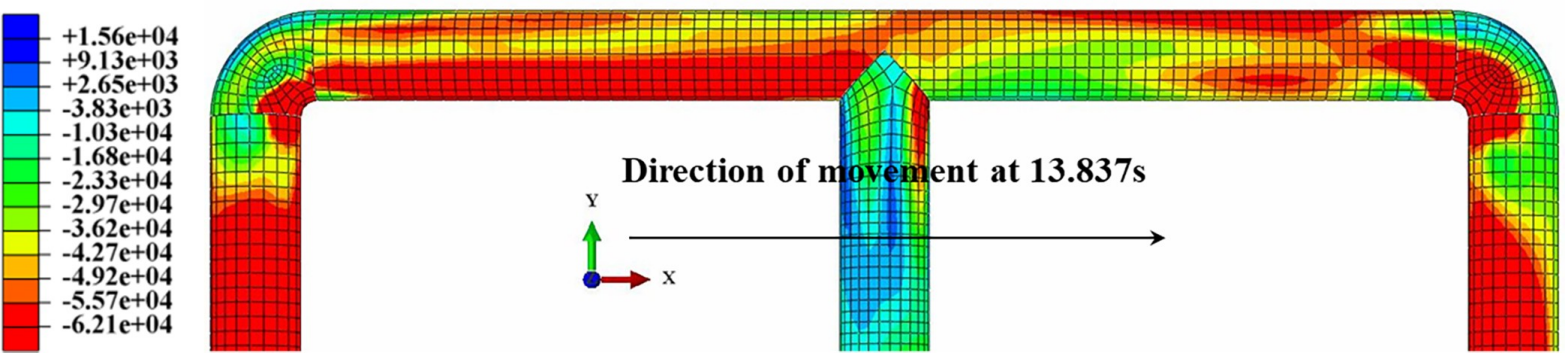
Axial strain contour of scale pipe network.

From Table 3, it can be found that although there were some differences between the results of the numerical simulation and the test results, the change trend was completely consistent; this was because the soil test is affected by human factors as well as the dispersion of the soil particles. The finite element method is an ideal numerical simulation method; therefore it is difficult to use the finite element method to accurately simulate the interaction between the soil particles and the pipe wall under real conditions; however the simulation results can be used as a reference for the test results. Fig 9 shows the axial strain contour of the scale pipe network. It can be seen that the response of the pipe network in sand was larger than that in clay. Moreover, axial strain state in the pipe embedded in sand is compressive (negative axial strains), as opposed to the pipe embedded in clay.

### Analysis of the axial friction of the pipe network

**Fig 10:**
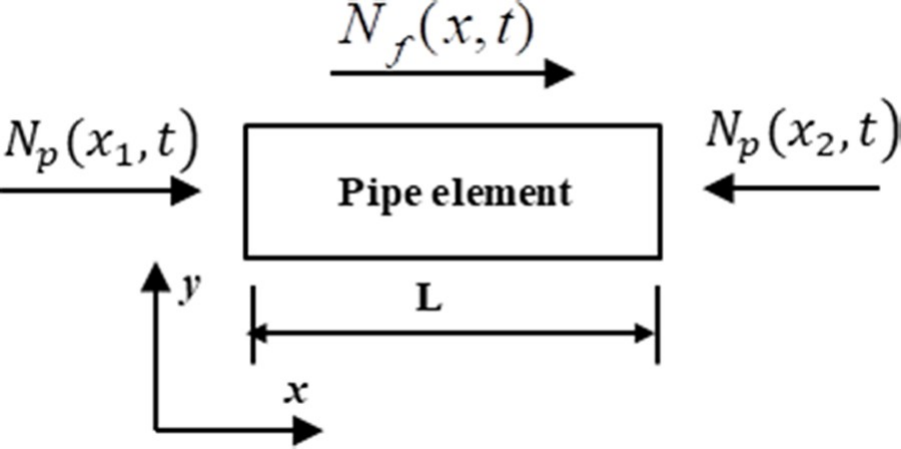
Force analysis of pipe element in *x* direction.

The value of the strain at 13.837 s was used to analyze the deformation and stress characteristics of the pipe network NP. The force diagram of the pipe element has been shown in Fig 10. The inertial force on the pipe was neglected and this satisfied the formula as follows Eqs (2)–(4):

$$N_{f}\left(x,t\right)=N_{p}\left(x_{2},t\right)-N_{p}\left(x_{1},t\right)$$ (2)

$$N_{p}\left(x_{1},t\right)=E\cdot A\cdot \varepsilon \left(x_{1},t\right)$$ (3)

$$N_{p}\left(x_{2},t\right)=E\cdot A\cdot \varepsilon \left(x_{2},t\right)$$ (4)

The friction force per unit length of pipe is:

$$f\left(x,t\right)=\frac{N_{f}\left(x,t\right)}{L}=\frac{E\cdot A\cdot (\varepsilon \left(x_{2},t\right)-\varepsilon \left(x_{1},t\right)}{L}$$ (5)

where *N*_*f*_(x,*t*) is the axial friction of the pipe element; *N*_*p*_(*x*_1_,t) is the axial force at the end *x*_*1*_ of the element; *N*_*p*_(*x*_2_,*t*) is the axial force at the end *x*_*2*_ of the element; *E* is the elastic modulus of the pipe; *A* is the cross sectional area of the pipe; *ε*(*x*_1_,*t*) is the axial strain at the end *x*_*1*_ of the element; *ε*(*x*_2_,*t*) is the axial strain at the end *x*_*2*_ of the element; *L* is the length of the pipe element; *f*(*x*,*t*) is the friction on per unit length of pipe.

**Fig 11:**
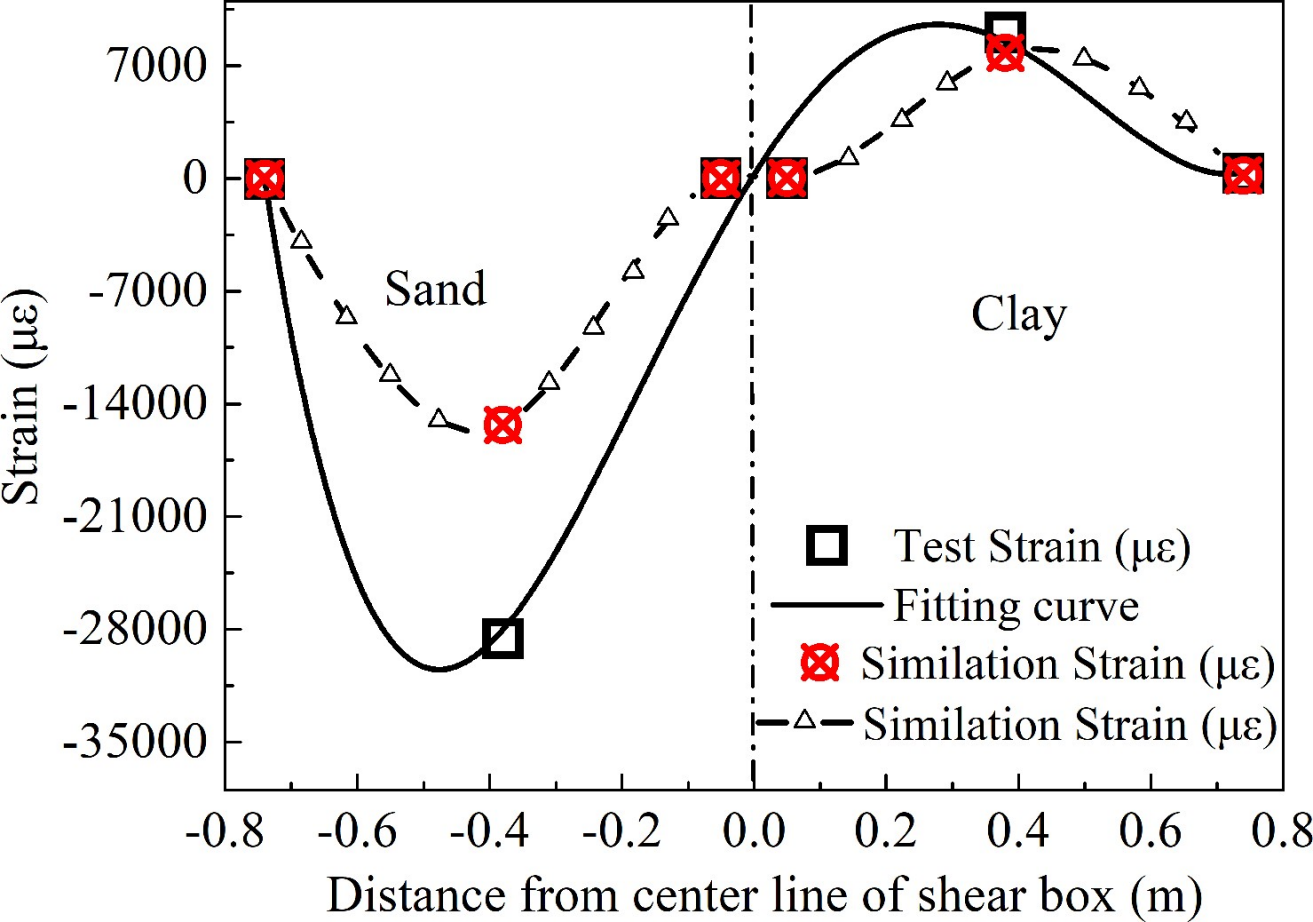
Fitting curve of axial strain for pipe network NP.

From Eq (5), it could be seen that the product of the slope of the curve fitted by the strain of the measuring point and the elastic modulus of the pipe and the cross-sectional area of the pipe was the friction of the pipe generated by the soil, and the fitting curve of the measuring point at 13.837 s for the pipe network NP has been shown in Fig 11.

The fitted curve was as follows in Eq (6), the correlation index was R^2^ = 0.9714, this value was greater than 0.8, therefore this has shown that the strain fitting curve met the engineering requirements:

$$S=171381x^{4}-118298x^{3}-94038x^{2}+65122x+239$$ (6)

where *S* is the axial strain of the pipe in με; *x* is the position coordinate of the pipe in *x* direction, in m.

**Fig 12:**
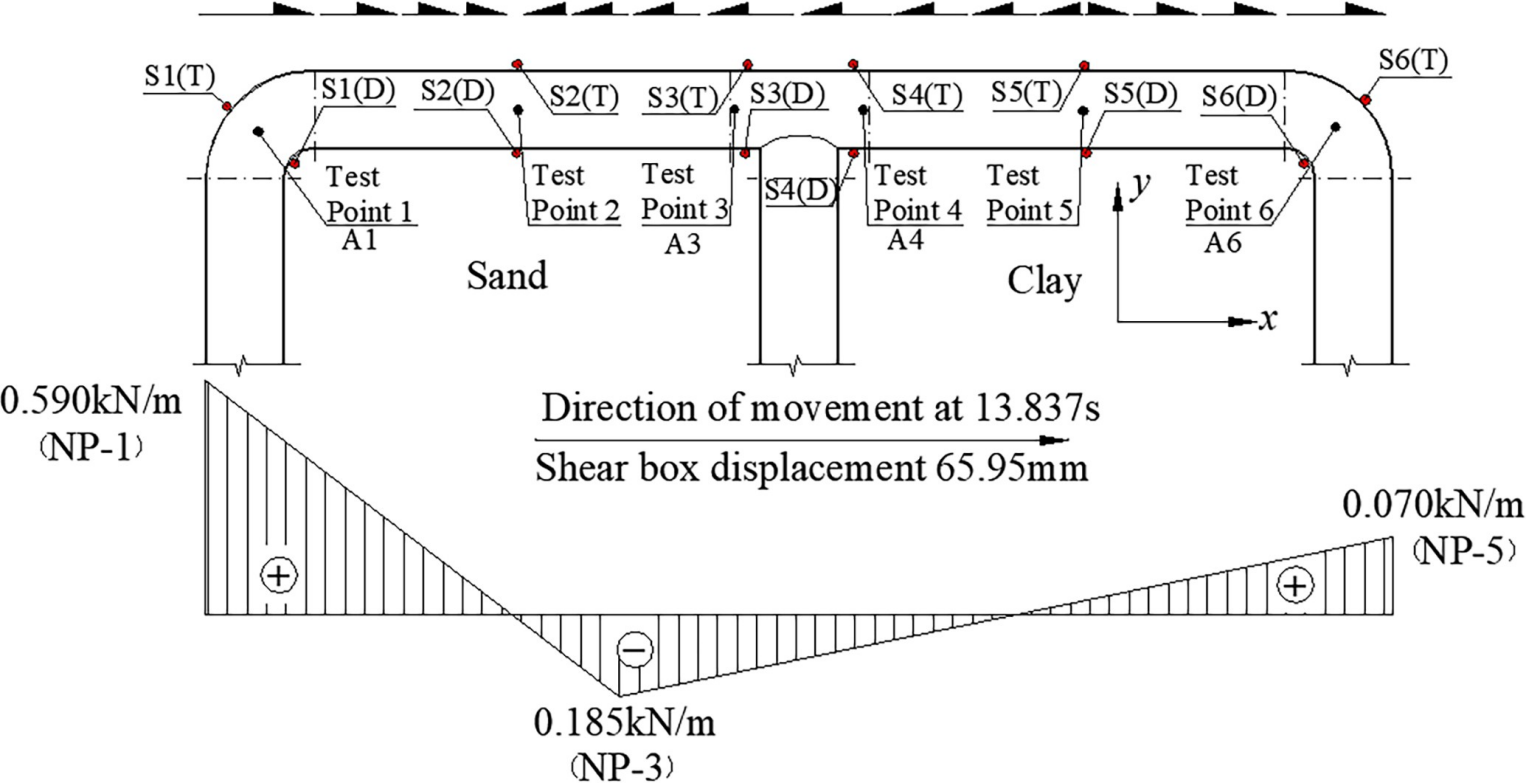
Pipe-soil friction along axial direction outside the pipe network NP.

**Fig 13:**
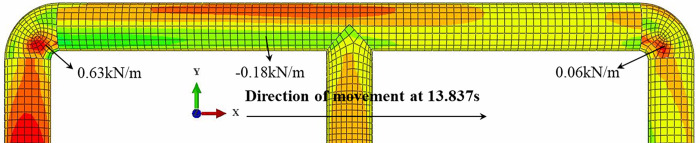
Contour of tangential contact force on the surface of scale pipe network.

Eq (6) above was derived in the *x* direction to calculate the friction acting on the pipe. The direction and magnitude of the friction have been shown in Fig 12, with inflection points in the direction of the friction near the midpoint of pipes NP-2 and NP-4. As the strain at the inflection point displayed an extreme value, the deformation of the pipe network should extend to the outside of the pipe network at this point; the friction at NP-1 and NP-5 was the largest, and were the same as the overall movement direction of the pipe; the friction at NP-3 had the largest value in the opposite direction, and it could be seen that the strain response of the sand was greater than that of the clay, and the response to the friction was also larger. This caused the friction to act to the left of the maximum point in the negative *x* direction. Fig 13 shows the contour of the contact friction of the pipe network. It can be seen that the calculation results of the numerical simulation are in good agreement with the test results, which confirms the correctness of the curve fitting results.

### Bending deformation of the pipe network

**Fig 14:**
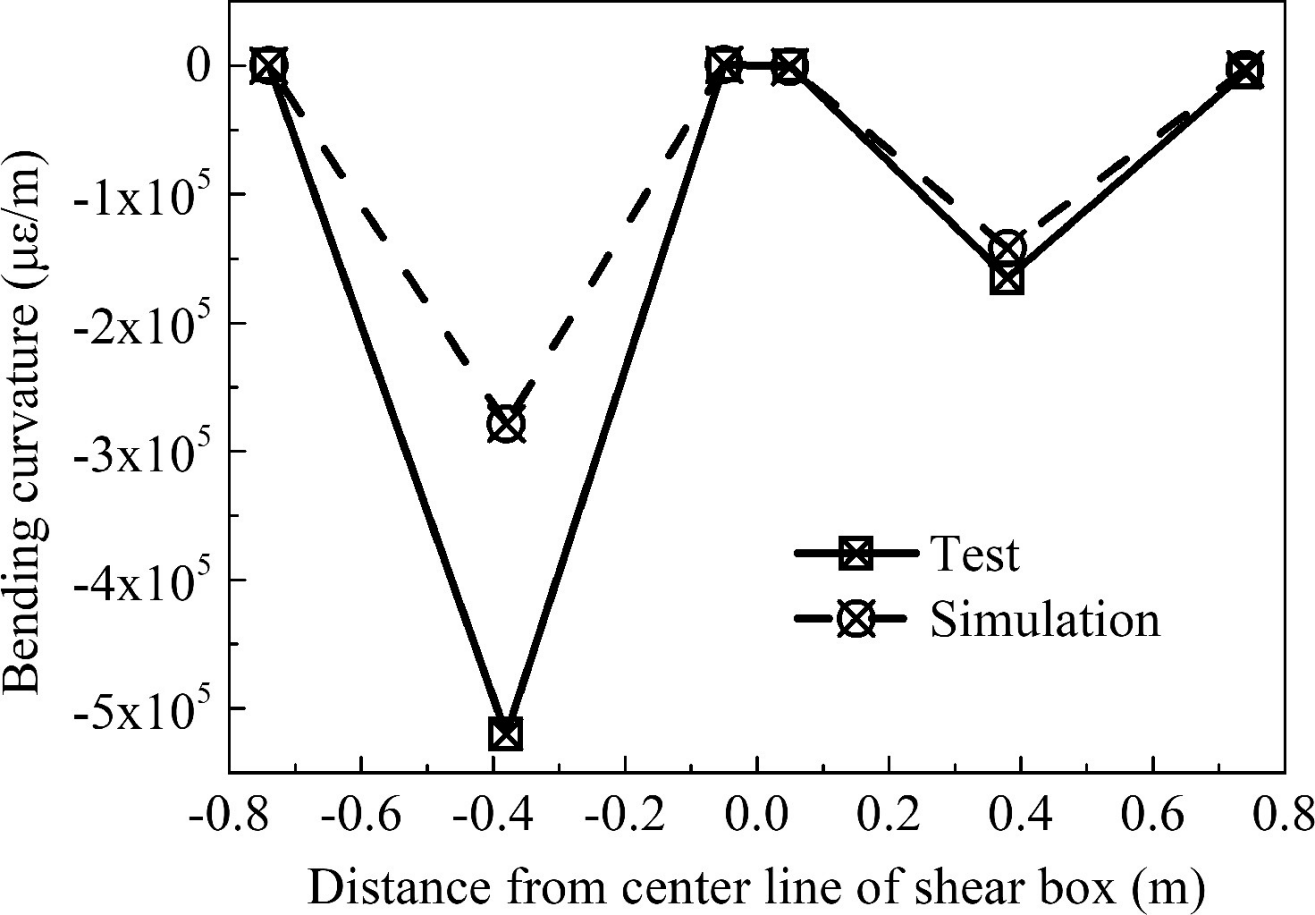
Graph of the bending curvature at the outside of pipe network NP.

The bending deformation of the pipe network is also an important index that can be used to study the response of a pipe network during an earthquake. The bending curvature was obtained from Eq (7), where *D* is the diameter of the pipe with a value of 0.11 m. Table 4 has shown the bending curvature of each test point of the pipe network NP. Fig 14 has shown the graph of the bending curvature of the pipe network NP. From the figure, it can be seen that the bending curvature of pipe embedded in sand was larger than that in clay. That is to say, the seismic response of the pipe network in sand was larger.

$$\phi_{p-n}=\frac{\varepsilon_{D-n}-\varepsilon_{T-n}}{D}$$ (7)

**Table 4:** Bending curvature of all test points for the pipe network.

Test point	Test Point 1	Test Point 2	Test Point 3	Test Point 4	Test Point 5	Test Point 6
**Test (με/m)**	242	-520000	318	-291	-164900	-5482
**Simulation (με/m)**	227	-278454	864	-754	-142054	-2690

### Deformation amplification coefficient of the pipe network nodes

**Fig 15:**
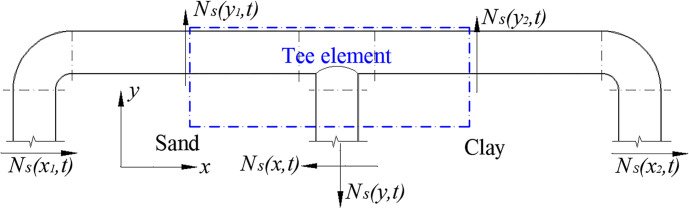
Deformation analysis of pipe network NP.

The relationship between node deformation and non-node deformation is able to reflect the seismic response of both the node and the pipe. The deformation of the elbow and the tee is the key to the deformation of the pipe network’s nodes. In this study, the ratio of the maximum strain and the maximum axial strain of the same cross-section measuring point was used as the deformation amplification coefficient *α*_*c*_ of the pipe network nodes. This was used to analyze the deformation characteristics of each type of joint of the pipe network during an earthquake. Table 5 has shown the deformation amplification coefficient of each measuring point of the pipe network NP. It can be seen from the table that the amplification coefficient of the tee was larger than that of the elbow. Moreover, the deformation amplification coefficient of Test Point 3 in sand was larger than Test Point 4 in clay, which indicated that the shear force of pipe NP-2 in sand was larger, that is to say, the deformation of NP-2 in sand was larger than that in clay. It can be seen from Fig 14 that the deformation of the pipe in sand was much larger than that in clay. As shown in Fig 15, due to the larger shear force *N*_*s*_(*y*_1_,*t*) of the tee element NP-2 and the larger bending moment at the tee joint, the deformation of the tee in sand was larger than that in clay. In contrast, for elbows, the deformation amplification coefficient of the elbows in clay was larger than that in sand, as shown in Fig 15, which meant that the shear force *N*_*s*_(*y*_2_,*t*) of a straight pipe that was perpendicular to the seismic wave in clay was larger than that in sand. The bending moment at the elbow in clay was larger than that in sand, so the deformation of the elbow in clay was larger than that in sand.

**Table 5:** Deformation amplification coefficient of all test points of the pipe network NP.

Test point	Coordinate (m)	*α*_*c*_	
		Test	Simulation
**Test Point 1**	-0.74	1.32	1.73
**Test Point 2**	-0.38	2.00	2.00
**Test Point 3**	-0.05	8.00	6.22
**Test Point 4**	0.05	2.14	2.52
**Test Point 5**	0.38	2.00	2.00
**Test Point 6**	0.74	1.97	1.89

The above analysis has shown that the deformation amplification coefficient of a tee was between 2.14 and 8.00, and that of an elbow was between 1.32 and 1.97, which is related to the properties of the soil. Liu and Hou [40] calculated the strain amplification coefficient for tees and elbows, *λ*, to be:

$$\lambda =\{\begin{array}{cc} 1.5 & \text{elbows}\\ 1.8 & \text{tees} \end{array}$$ (8)

During loading, due to the mutual restriction between the pipes in the pipe network, the edge shear force and the edge moment were generated at the connections of the pipes in order to maintain the same connections as those before deformation; that is, deformation coordination of the pipe network was achieved, as a result of this the deformation amplification coefficient was too large.

### Pipe-soil relative displacement of pipe network

**Fig 16:**
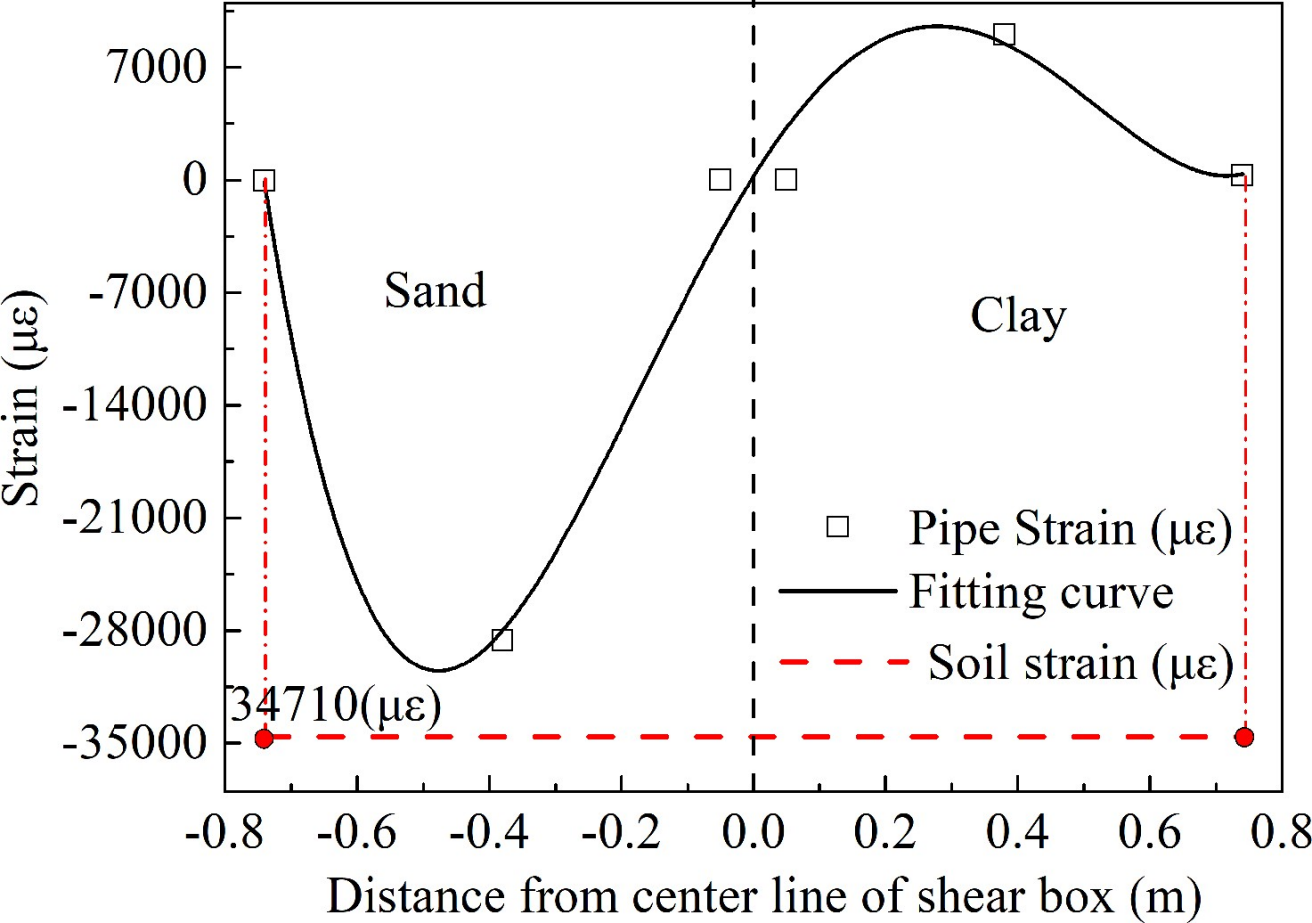
The strain curve of soil and pipe network NP.

The displacement of the shear box was 65.95 mm at 13.837 s; due to the inertia the displacement of the shear box was regarded as deformation of the soil, which was compression deformation. The strain in the soil around the pipe was *ε* = 65.95/1900 = 0.03471 = 34710 με > > 100 με as shown in Fig 16. Since the strain of the soil was much larger than 100 με at this time, there was relative displacement between the soil and the pipe [41]. That is to say, the displacement of the pipe was not consistent with that of the soil. At this time, the displacement of the soil was larger than that of the pipe. The pipe-soil relative displacement can be calculated by subtracting the deformation of the soil from the deformation of the pipe.

The fitting curve of the strain at the measuring point of the pipe network NP was integrated in the *x* direction in order to obtain the deformation of the pipe network NP, which was the relative displacement from the elbow point NP-1 to the elbow point NP-5, as shown in Eq (9) below. The deformation of the pipe from NP-1 to NP-5 in the network NP was then calculated to be 49.19 mm:

$$D_{p}=\int_{-0.74}^{0.74}\left(171381x^{4}-118298x^{3}-94038x^{2}+65122x+239\right)\times 10^{-3}\text{d}x=49.19mm$$ (9)

From Table 6, the relationship of the relative displacement between the pipe network NP and the soil can be seen. At 13.837 s, the total axial deformation *D*_*p*_ of the NP in *x* direction was 74.6% of the deformation *D*_*G*_ of the soil in *x* direction, and the relative displacement was 25.4% of the deformation of the soil. In other words, about 25% of the deformation energy of the soil was transferred into the internal force and deformation of the pipe during the earthquake and that the larger the ratio of the relative displacement was, the more unfavorable the deformation of the pipe was.

**Table 6:** Deformation relationship between the pipe network NP and the soil.

Groups	*D*_*G*_ (mm)	*D*_*p*_ (mm)	*D*_*p*_/*D*_*G*_	(*D*_*p*_/*D*_*G*_)/*D*_*G*_
**Test**	65.95	49.19	74.6%	25.4%
**Simulation**	60.32	48.25	80.0%	20.0%

**Fig 17:**
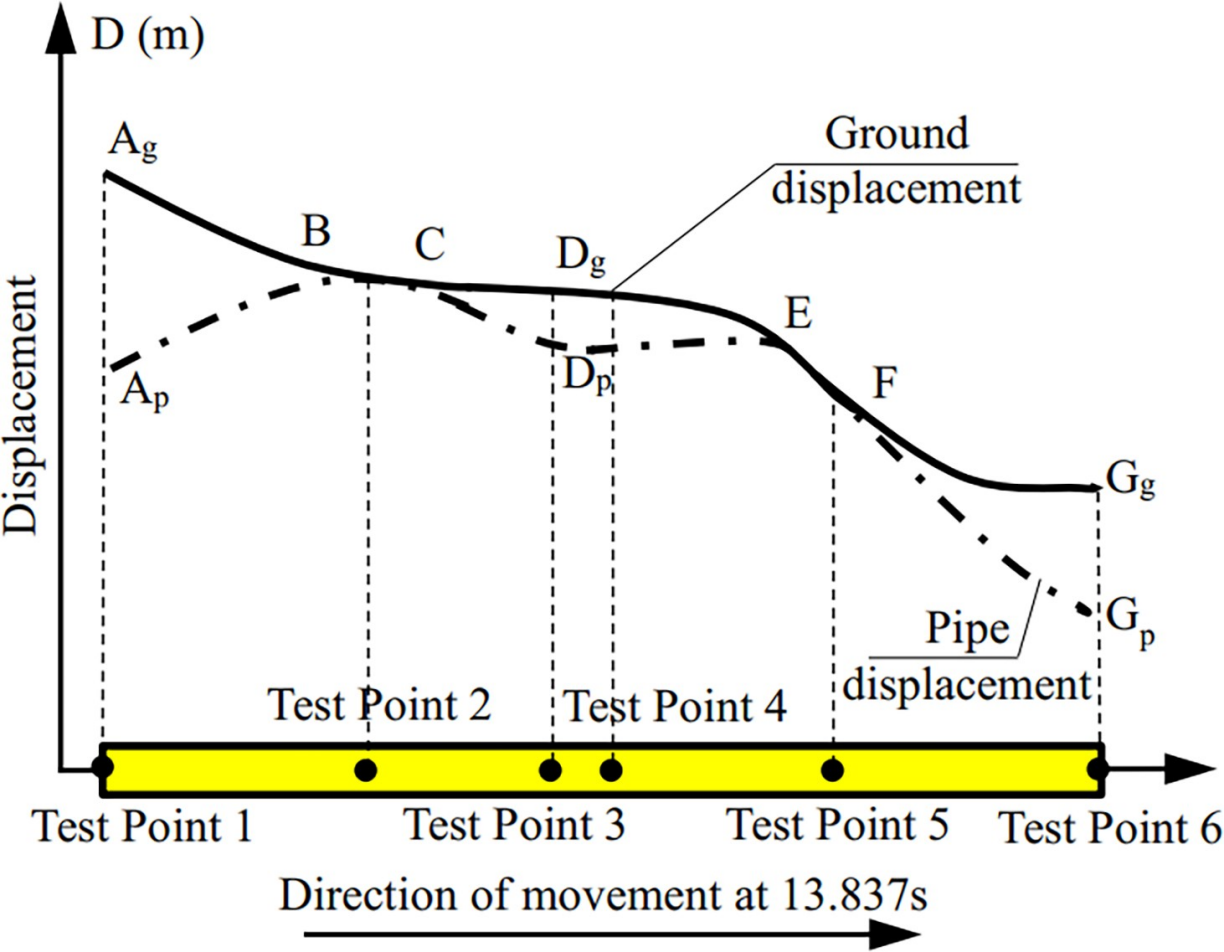
Diagram of displacement relationship between pipe network NP and soil.

According to the above data, the graph in Fig 17 could be obtained; Fig 17 has shown the relationship between the pipe displacement and soil displacement for the pipe network NP. The soil displacement from A_g_ to G_g_ was on the upper side of the pipe displacement from A_p_ to G_p_. This figure is also a response to the pipe’s friction, that is to say, the friction gradually decreased from A_p_ to B, and the friction was close to 0 from B to C, which was the midpoint of NP-2. D_p_ was near the tee, E to F was near NP-4, F to G_p_ was the right half of NP-4 with increased friction.

The transfer coefficient of pipe deformation and soil deformation was given in the Chinese code [42], which has been denoted *ξ* in the following Eqs (10)–(12):

$$D_{p}=\xi D_{G}$$ (10)

$$\xi =\frac{1}{1+{\left(\frac{2\pi }{\lambda }\right)}^{2}\frac{EA}{k}}$$ (11)

$$\lambda =v\cdot T_{g}$$ (12)

where *v* is the shear wave velocity of soil; *T*_*g*_ is the characteristic period of soil; *E* is the elastic modulus of the pipe; *A* is the cross-sectional area of the pipe; *k* is the elastic stiffness of the spring representing the pipe-soil interaction in the longitudinal direction. According to the corresponding determination,*v* = 200 m/s, *T*_*g*_ = 0.5 s, *E* = 4000 MPa, $A=\frac{\pi \left(110^{2}-106.8^{2}\right)}{4}=545\;{\text{mm}}^{2}$. As for the pipe-soil contact spring stiffness, it was *k* = 2×10^6^ N/m, detailed calculation instructions have been provided in the literature [2]. According to the calculation, *ξ* = 0.699, for the test conditions of this study that value was *ξ* = 0.746, so the value used in the code was more conservative. Due to the large ratio of the pipe-soil relative displacement in this study to the displacement of the soil in the code, which was unfavorable to the response of pipe, the calculation formula in the code is safer for a pipe.

## Test results and analysis of the H-shaped and Z-shaped pipes

### Strain test results of the H-shaped and Z-shaped pipes

For the H-shaped pipe, the maximum value of the displacement of the soil DS in the shear box of the shaking table was 61.307 mm, and the time that corresponded to the maximum displacement was 15.042 s; therefore the selection time that corresponded to the strain was 15.042 s. For the Z-shaped pipe, the maximum value of the displacement of the soil DS in the shear box of the shaking table was 61.088 mm, and the time that corresponded to the maximum displacement of the soil was 8.451 s; therefore the selection time that corresponded to the strain was 8.451 s. The measured strain result, numerical simulation result and the calculated axial strain value of each test point have been shown in Table 7.

**Table 7:** Measured strain and calculated axial strain of H-shaped and Z-shaped pipes.

Sensor number	Measured value (με)				Coordinate (m)	Axial strain (με)			
Shape	H		Z		H/Z	H		Z	
	Test	Simulation	Test	Simulation		Test	Simulation	Test	Simulation
**S1(T)**	-31	-37	-35	-30	-0.52	-62	-64	-1021	-1066
**S1(D)**	-93	-90	-2006	-2101					
**S2(T)**	-8	-13	-18	-23	0	-3	-5	-6	-7
**S2(D)**	2	4	6	9					
**S3(T)**	-3	-6	-6	-10	0.52	-32	-39	-25	-30
**S3(D)**	-60	-72	-43	-49					

### Analysis of the axial friction for the H-shaped and Z-shaped pipes

**Fig 18:**
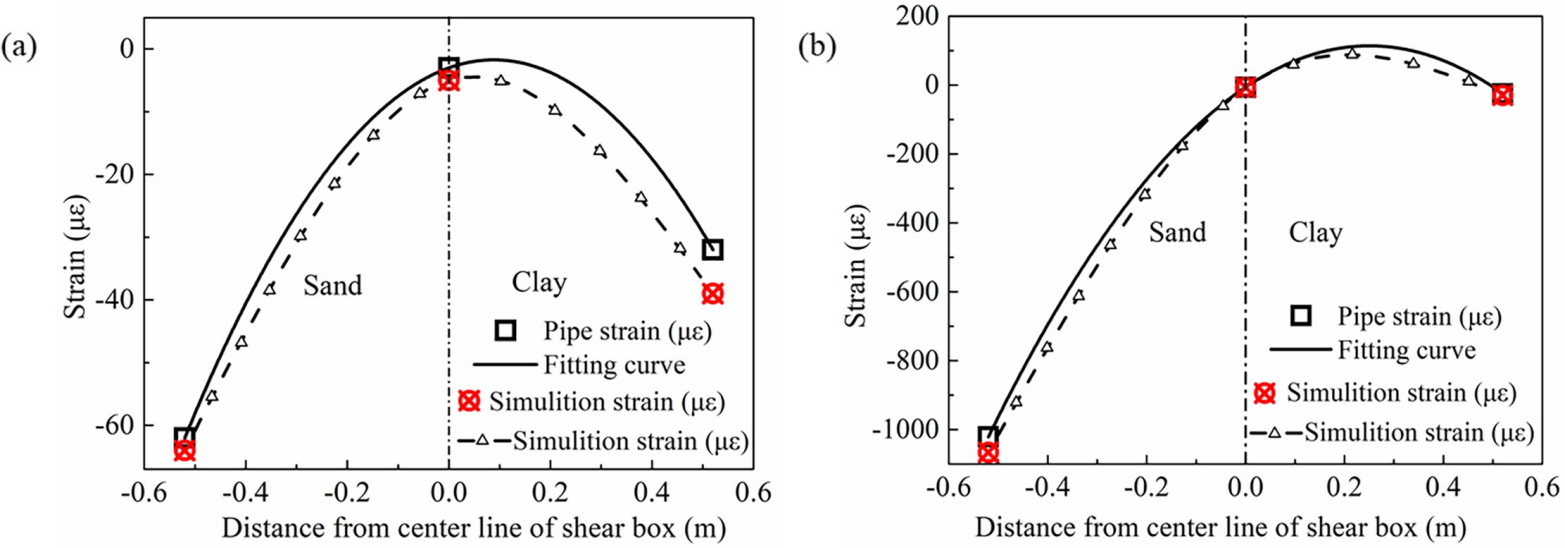
Strain fitting curves of test points for H-shaped and Z-shaped pipes. (a) H-shaped pipe, (b) Z-shaped pipe.

**Fig 19:**
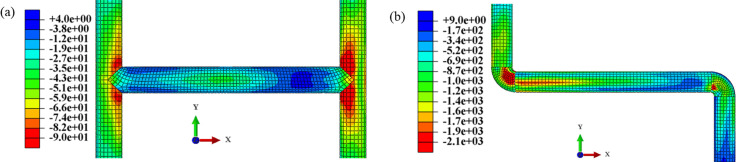
Axial strain contour of H-shaped and Z-shaped pipes. (a) H-shaped pipe, (b) Z-shaped pipe.

Fig 18A has shown the fitting curve for the axial strain of the H-shaped pipe, and Fig 18B has shown the fitting curve for the axial strain of the Z-shaped pipe. Fig 19 has shown the strain contour of numerical simulation of the H-shaped pipe and the Z-shaped pipe. The feasibility of the test can be seen by comparing the test results with the numerical simulation results. From the Figs 18 and 19, it can be seen that the response of sand is still larger than that of clay. Since the Z-shaped pipe only had elbows in it, the deformation could only be transmitted to the straight pipe. However, the H-shaped pipe joint was a tee, and the deformation on either side of the same tee can restrict each of the sides, therefore the Z-shaped pipe responded more violently to the deformation than the H-shaped pipe. In comparison with Fig 11, it can be found that the strain of the pipe network NP in sand and clay had extreme points due to the tee NP-3 of the pipe network NP; this shows that the addition of a tee at the midpoint of a straight pipe can weaken the response to the deformation of the pipe. The following Eqs (13) and (14) describe the fitting curves of the axial strain of the two pipes. In comparison with Eq (6), it can be seen that the maximum power of the fitting results can be increased by 2 when the tee NP-3 was added in the middle of the pipe network. From these equations it can be seen that there will be two extreme points in both the sand and the clay; this has shown that the safety of straight pipes on both sides of a tee has been improved:

$$S_{H}=-162.72x^{2}+28.85x-3$$ (13)

$$S_{Z}=-1911.98x^{2}+957.69x-6$$ (14)

**Fig 20:**
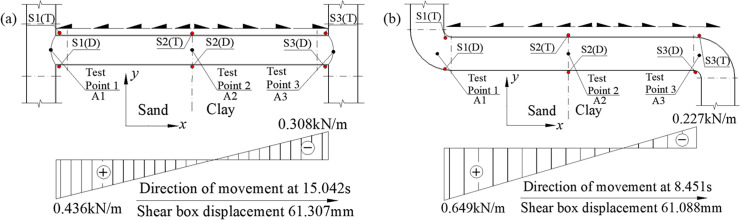
Friction between soil and pipe along the seismic direction for H-shaped and Z-shaped pipes. (a) H-shaped pipe, (b) Z-shaped pipe.

**Fig 21:**
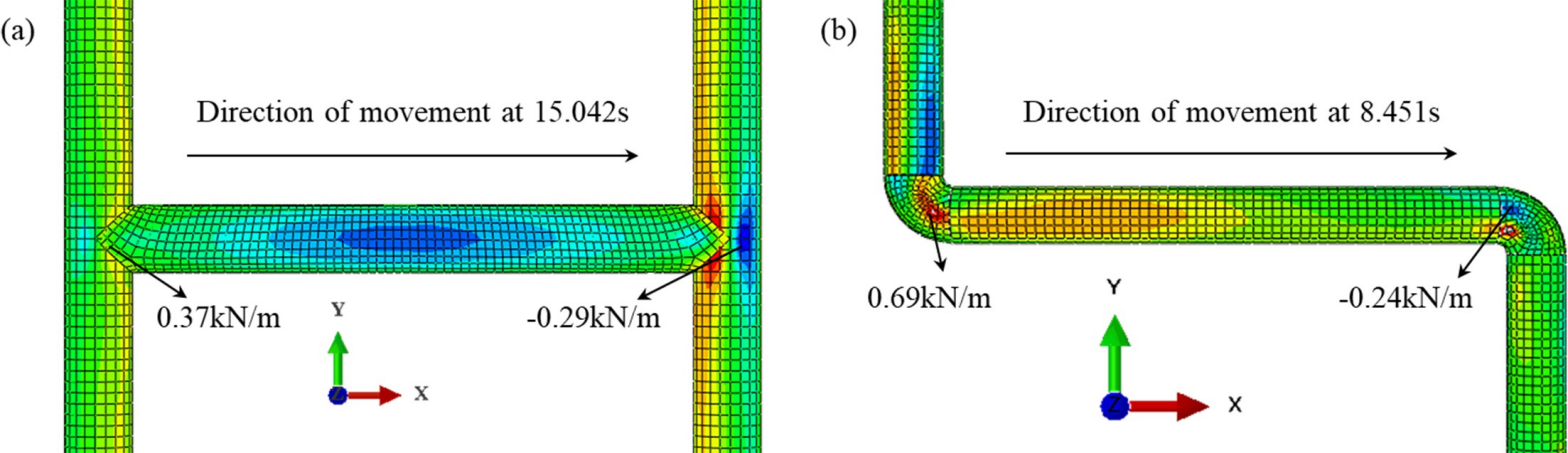
Contour of tangential contact force on the surface of H-shaped and Z-shaped pipes. (a) H-shaped pipe, (b) Z-shaped pipe.

Fig 20A and 20B has shown the friction values of the H-shaped and Z-shaped pipes under the soil using the axial strain of the pipes. Fig 21A and 21B has shown the contour of pipe-soil contact friction. From the Figs 20 and 21, it can be seen that the friction response of the sand was greater than that of the clay, and the friction at the inner arc of the elbow was greater than that at its outer arc. There was no outer arc on the tee, therefore the friction of the H-shaped pipe for both the sand and the clay was not very different, while the friction for the sand and clay of the Z-shaped pipe was significantly different. From comparison with Fig 12, it can be seen that the tee NP-3 could also effectively change the direction of the friction and reduce the response of the pipe network.

### Bending deformation of the H-shaped and Z-shaped pipes

**Fig 22:**
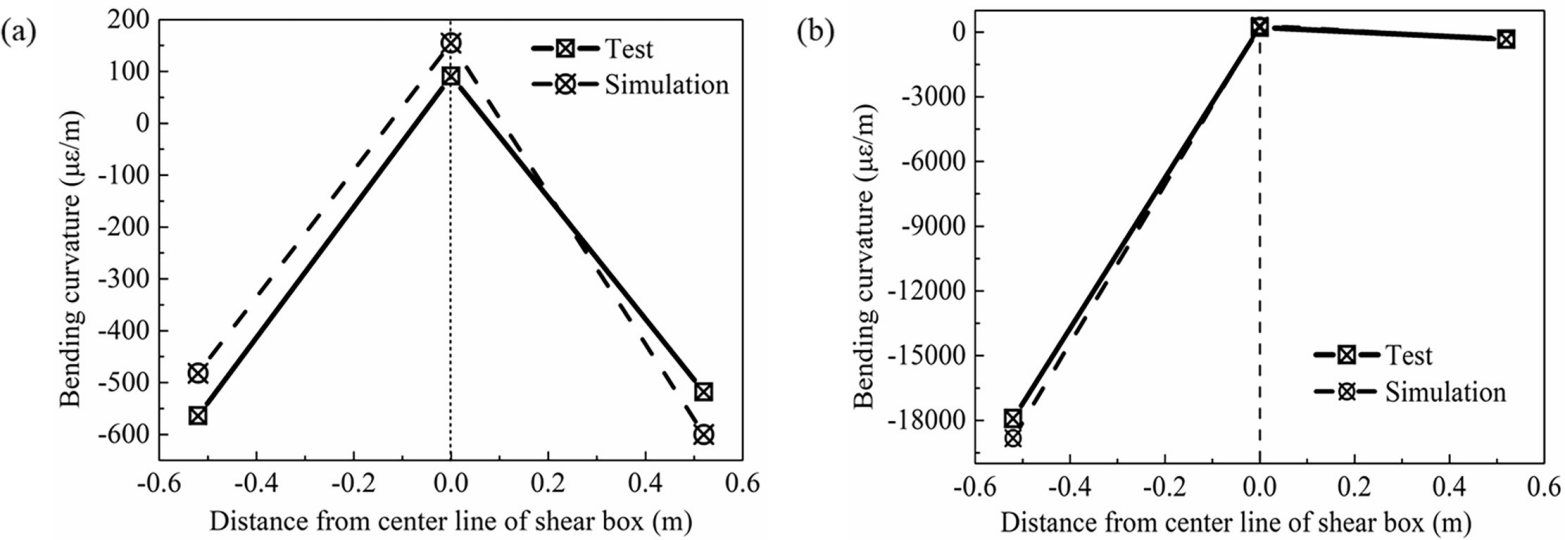
Graph of the bending curvature of H-shaped and Z-shaped pipes. (a) H-shaped pipe, (b) Z-shaped pipe.

Table 8 has shown the bending curvature of the H-shaped and Z-shaped pipes. It can be seen from Fig 22 that the bending curvature of the two tees of the H-shaped pipe (Fig 22A) was similar. Since the Z-shaped (Fig 22B) pipe is not axisymmetric, the bending curvature of the elbow in the sand was greater for the movement direction at 8.451s. Compared with Fig 14, the tee NP-3 in the pipe network NP produced two extreme points in the bending deformation curve, which then resulted in the pipe network NP reaching deformation coordination.

**Table 8:** Bending curvature of H-shaped and Z-shaped pipes.

Pipe type	Test point	Test (με/m)	Simulation (με/m)
**H-shaped**	Test Point 1	-564	-482
	Test Point 2	91	155
	Test Point 3	-518	-600
**Z-shaped**	Test Point 1	-17918	-18827
	Test Point 2	218	291
	Test Point 3	-336	-355

### Deformation amplification coefficient of the H-shaped and Z-shaped pipes

Table 9 has shown the deformation amplification coefficient of each test point of the H-shaped and Z-shaped pipes. From the table it can be found that the deformation amplification coefficient of the H-shaped pipe was between 1.50 and 1.88, and that of the Z-shaped pipe was between 1.72 and 1.96; therefore the deformation amplification coefficient of the tee was almost the same as that of the elbow. Compared with Table 5, the deformation amplification coefficient of tee NP-3 was significantly increased; this indicated that adding a tee to a straight pipe will aggravate the degree of uneven deformation of the tee and can easily lead to damage to the joints. The deformation amplification coefficient of both the elbows NP-1 and NP-5 in the pipe network was between 1.32 and 1.97, which included the elbow amplification coefficient of the Z-shaped pipe; this indicates that the degree of uneven deformation of the elbow in the pipe network will not increase due to the mutual coordination of the pipes. According to Hall and Newmark’s [43] experiments on thin-walled cylinders, wrinkling in pipes occurs at the strain in the following range:

$$0.15\cdot t/R\leq \varepsilon_{cr}\leq 0.20\cdot t/R$$ (15)

where *t* and *R* are the wall thickness and radius of the pipe, respectively. Therefore, the theoretical local buckling strain in the tee NP-3 was about 8000 *με*, while the compressive strain of the tee in the test was 40 *με*; therefore this is far from this degree of buckling. However, during the test, the connection between NP-2 and NP-3 slipped.

**Table 9:** Deformation amplification coefficient of two kinds of pipe joints.

Pipe type	Test point	Coordinate (m)	*α*_*c*_	
			Test	Simulation
**H-shaped**	Test Point 1	-0.52	1.50	1.41
	Test Point 2	0	2.67	2.60
	Test Point 3	0.52	1.88	1.85
**Z-shaped**	Test Point 1	-0.52	1.96	1.97
	Test Point 2	0	3	3.29
	Test Point 3	0.52	1.72	1.63

### Pipe-soil relative displacement of the H-shaped and Z-shaped pipes

The fitting curves of the strain at the test points of the H-shaped and Z-shaped pipes were then integrated in the *x* direction in order to obtain the relative displacement of the test points at the ends of the H-shaped and Z-shaped pipes, as shown in Table 10 below. The difference between the deformation of the pipes and the soil for the H-shaped or Z-shaped pipes accounted for 30.0% and 30.4% of the deformation of the soil respectively (27.9% and 27.5% from simulation). In comparison it can be seen from Table 6 that the pipe-soil relative displacement of the pipe network NP was smaller than that of a single pipe. It is obvious that the soil action of a single pipe was large, but that the deformation energy of the pipe could easily be released. Due to the mutual constraint caused by the deformation of the pipe network, it is difficult to release the energy. As a result, shear force and a bending moment will be produced at the end of the tee and elbow, and the destruction of the pipe network’s nodes will be more serious. It can be seen from last section that this damage mainly manifested as damage to the nodes’ connections. Due to an increase in the number of the tee or elbow joints in a pipe network, the proportion of the pipe-soil relative displacement will decrease.

**Table 10:** Deformation and relative displacement of H-shaped and Z-shaped pipes.

Pipe type	Groups	Time (s)	*D*_*G*_ (mm)	*D*_*p*_ (mm)	*D*_*p*_/*D*_*G*_	(*Dp*/*D*_*G*_)/*D*_*G*_
**H-shaped**	Test	15.024	61.307	42.93	70.0%	30.0%
	Simulation	15.024	58.296	42.01	72.1%	27.9%
**Z-shaped**	Test	8.451	61.088	42.54	69.6%	30.4%
	Simulation	8.451	57.763	41.87	72.5%	27.5%

### Influence of elbows and tees joint on response of pipe network

When an underground pipe branches at an intersection, a tee will be added to the straight pipe section of the pipe network. When a straight road becomes a curved road, elbows will be added to the pipe network underneath the road. The seismic strain response after the addition of the tee will make two extreme points appear on either sides of the tee, so that the strain of the pipe will not increase infinitely; this is conducive to the safety of straight pipes in the pipe network. Moreover, the tees will also change the friction direction between the soil and the pipes, so that the friction at the pipe network will tend to reduce. The existence of a tee will make the deformation of straight pipes on both sides of a tee display extreme points in the pipe network, and thus improve the safety of straight pipes. However, due to the existence of a tee in the pipe network, this will aggravate the degree of uneven deformation of the tee, while an elbow set in the pipe network will not aggravate the degree of uneven deformation of an elbow in the case of deformation of a single pipe.

An increase in the numbers of tees or elbows in a pipe network will reduce the relative displacement between the pipe network and the soil. Although from the surface, with the decrease in the relative displacement, the seismic response of the pipe network will also decrease, due to the mutual restriction of the deformation among the pipes in the pipe network, and the response of each pipe in the pipe network was often more severe than that of a single type of pipe in the soil, and the damage that resulted at the nodes was also serious.

With the increase of the number of elbows or tee joints in the pipe network, the discoordination of the deformation of the pipes will increase, and the force between the pipes and the nodes will increase; this means that the connections between the nodes will be more easily damaged. The results from the above research can provide guidance for the layout and design of an underground pipe network.

## Conclusion

In this paper, through the use of shaking table tests for a scale pipe network, H-shaped and Z-shaped pipe network components, the response of the pipe network during an earthquake and the influence of tees and elbows on the response of pipe network have been obtained. The following conclusions could be drawn:

- For the pipe network, the extreme values of strain are produced between a tee and an elbow, where the pipe-soil friction is the smallest and the friction at the tee is the largest. The response of sand to axial strain was greater than that of clay; inhomogeneous geology will also change the direction of the friction.
- The bending deformation of a tee in the pipe network was larger than that of an elbow, and the deformation amplification coefficient of an elbow in sand was larger than that in clay. For the relative displacement between the pipe network and the soil, most of the displacement of the soil was transferred into the displacement of the pipe, and the remaining small part was transferred into the deformation energy of the pipe network. The relative displacement between the pipe network and the soil was also a response to the friction between the pipe and the soil.
- The strain and deformation of the straight pipes on both sides of the tee in pipe network have displayed extreme points, which resulted in the straight pipes in the pipe network tending to be safe. Moreover, the pipe network will aggravate the degree of uneven deformation of the tee. An increase in the number of elbows or tees in a pipe network will reduce the relative displacement between the pipe network and the soil, but this will lead to an increase in the discoordination of the deformation of the nodes of the pipe network. The tee and elbow joints are more likely to be destroyed.

## Supporting information

S1 Table Strain of the scale pipe network and displacement of shear box in test. (XLSX)

S2 Table Strain of the H-shaped pipe and displacement of shear box in test. (XLSX)

S3 Table Strain of the Z-shaped pipe and displacement of shear box in test. (XLSX)

## Abbreviations

A: Section area of pipe
D: Outside diameter of pipe
D_G_: Deformation of soil
D_p_: Deformation of pipe
E: Elastic modulus of pipe
*f*(*x*,*t*): Friction of pipe per unit length
k: Contact spring stiffness between soil and pipe
L: Length of pipe element (m)
*N*_*EH*_ (*N*_*EZ*_): Integration of Earth pressure for H-shaped pipe (Z-shaped)
*N*_*ES*_ (*N*_*EC*_): Integration of earth pressure of pipe network NP in sand (clay)
*N*_*f*_(*x*,*t*): Axial friction of pipe element
*N*_*p*_(*x*_1_,*t*)(*N*_*p*_(*x*_2_,*t*)): Axial force of pipe element at *x*_1_ at time *t* (*x*_2_)
*N*_*S*_(*y*_1_,*t*)(*N*_*S*_(*y*_2_,*t*)): Shear force of pipe element at *y*_1_ at time *t* (*x*_2_)
S: Strain of pipe network NP (με)
*S*_*H*_ (*S*_*Z*_): Strain of H-shaped pipe (Z-shaped) (με)
T_g_: Characteristic period of soil (s)
x: Distance from the center line of the shear box (m)
*ε*_*x*−*n*_: Axial strain of pipe section n
*ε*_*T*-*n*_ (*ε*_*D*-*n*_): Axial strain for test point at the top of pipe section n (at the down of pipe section n)
*ε*(*x*_1,_*t*)(*ε*(*x*_2_,*t*)): Axial strain of pipe element at *x*_1_ at time *t* (*x*_2_)
ε: Strain of soil
*ϕ*_*p*-*n*_: Bending curvature of pipe section n (με/m)
λ: Strain amplification coefficient of pipe joint
ξ: Deformation transfer coefficient between pipe and soil
v: Shear wave velocity of soil
